# Microwave-Assisted Digestion of Polyurethane Foam as an Alternative to Elution: Solid Phase Extraction of Cd(II) and Pb(II) for Their Determination in Swimming Pool Waters

**DOI:** 10.1155/2023/9624637

**Published:** 2023-05-30

**Authors:** Juliana Menezes de Sousa, Graziela Fregonez Baptista Cruz, Luiza Gomes dos Santos, Ricardo J. Cassella

**Affiliations:** Department of Analytical Chemistry, Fluminense Federal University, Outeiro de São João Batista s/n Centro, Niterói, RJ 24020-141, Brazil

## Abstract

In this work, a separation/preconcentration method is proposed for the determination of Cd(II) and Pb(II) in swimming pool waters, using ammonium pyrrolidine dithiocarbamate (APDC) as a complexing agent and unloaded polyurethane foam (PUF) as a sorbent. The proposed method was optimized, and the defined optimal conditions were a pH of 7, 30 min of shaking time, 400 mg of PUF, and 0.5% (m/v) of the APDC solution. The release of Cd(II) and Pb(II) from the solid phase was achieved through the total digestion of PUF using a microwave-assisted acid approach with a 10.5 mol·L^−1^ HNO_3_ solution. The methodology was applied to four samples of swimming pool water for the determination of Cd(II) and Pb(II) using graphite furnace atomic absorption spectrometry (GF AAS). The limits of detection and quantification obtained were 0.02 and 0.06 *μ*g·L^−1^ for Cd(II) and 0.5 e 1.8 *μ*g·L^−1^ for Pb(II), respectively. We analyzed four samples of swimming pool waters, finding Cd concentrations between 0.22 and 1.37 *μ*g·L^−1^. On the other hand, only one sample presented Pb concentration above the limit of quantification (11.4 *μ*g·L^−1^). Recovery tests were performed by spiking the samples with known concentrations of the analytes, and recovery percentages between 82% and 105% were obtained.

## 1. Introduction

The use of swimming pools has increased throughout the years, namely, for sports, leisure, and/or therapy as a result of an increase in consciousness regarding the social, physical, and psychological importance of swimming. As a result, ensuring that swimming pool waters exhibit a suitable quality has become increasingly important to avoid risks to the health of swimmers. Indeed, new technologies are currently being developed to minimize the eventual negative effects of swimming pool waters on public health, including the development of analytical tools for water quality monitoring methods [1, 2]. In this sense, the determination of the concentration of metals in the waters is important, especially due to their toxicity and capacity to cause adverse effects to biological systems which are directly associated with the amount and duration of exposure [3]. It is important to highlight that according to Ojezele et al. [4], skin absorption is the main source of metal toxicity in humans. Therefore, the determination of metals in swimming pools' water is a very important issue. In the cases of Cd and Pb, it was already demonstrated their absorption by the skin as an important pathway to human contamination [5, 6].

Lead and Cd do not have any essential metabolic functions for human beings. Lead is easily and rapidly distributed through different tissues and trespasses encephalic and placental barriers. It is toxic to our central and peripheral nervous systems and also causes renal and hematopoietic problems [7, 8]. Cadmium has been recognized to cause lung and prostate cancer, in addition to kidney failure, hypertension, infertility, and bone diseases [9].

In Brazil, the quality of swimming pool waters is primarily regulated by ANVISA (Brazilian Health Regulatory Agency), which establishes that certain conditions must be met to ensure the safe use of the former. However, ANVISA regulations have mainly focused on physical-chemical parameters, such as pH, clarity, and others, and nothing is mentioned regarding the concentration of metals. In turn, CONAMA (Brazilian National Environment Council) is responsible for the regulations of all types of water, depending on their use. The CONAMA law 357/2005 (March 17^th^, 2005) establishes that waters used for leisure purposes, such as those used for swimming, water-skiing, and diving, must not contain any more than 10 *μ*g·L^−1^ and 1 *μ*g·L^−1^ of Pb and Cd, respectively [10].

The possibility of using unloaded polyurethane foam (PUF) in sorption studies with no chemical treatment has allowed several studies on the preconcentration of metals for their determination by atomic spectrometric techniques. However, the retention of metallic cations by the foam without functionalization, at first, depends on the formation of neutral or anionic [11] compounds. Some carbamates, including ammonium pyrrolidine dithiocarbamate (APDC), have already been used for this purpose [12–14]. Indeed, the use of adequate organic ligands allows the formation of species in a water solution with a high affinity for PUF [15].

In the development of the proposed method, APDC was chosen as a complexing agent given our knowledge of its capacity for the complexation of Pb(II) and Cd(II). In addition, the strategy of metal elution was based on the total acid digestion of the solid phase, since the PUF can be easily destroyed in a microwave oven with a diluted nitric acid solution [16]. It is important to note that this procedure is a good alternative to conventional elution since PUF can be found on the market at a low cost. Moreover, the total digestion of the solid phase in a sealed flask ensures the total release of the analytes to the final digestate without their loss. Therefore, this work aimed to develop a method for determining the Pb and Cd concentrations in swimming pool waters samples by graphite furnace atomic absorption spectrometry (GF AAS) and PUF as a sorbent, following the release of analytes from the solid phase through a microwave-assisted acid digestion procedure.

## 2. Experimental Setup

### 2.1. Apparatus

A Varian model AA240FS (Mulgrave, Victoria, Australia) flame atomic absorption spectrometer was used in the optimization studies of the method. Cadmium and Pb hollow cathode lamps were run at 4.0 mA and 10.0 mA currents, respectively. The Cd signals were measured at 288.8 nm using a spectral bandwidth of 0.5 nm, whereas the Pb signals were measured at 217.0 nm using a spectral bandwidth of 1.0 nm. An air (13.5 L·min^−1^)/acetylene (2.0 L·min^−1^) flame was used for the atomization of the analytes, and the background correction was performed with a deuterium lamp.

The determination of the analytes in the digestates was carried out with a Varian (Mulgrave, Australia) model AA240Z graphite furnace atomic absorption spectrometer, equipped with an autosampler Varian PSD 120, a Varian GTA 120 atomization unit, and a Zeeman-effect background correction system. The atomization was performed on graphite tubes with the L´vov platform, also supplied by Varian (Mulgrave, Victoria, Australia). The instrumental conditions (lamp current, wavelength, and spectral bandwidth) were the same as those used in the flame atomic absorption measurements. The analytical signals were computed as integrated absorbance values, and argon (99.99% purity) was used as the protective gas, which was supplied by Linde Gases (Macaé, Brazil).

The digestion of loaded foams was carried out with a SpeedWave Four®, Microwave Digestion System from Berghof (Baden-Württemberg, Germany), equipped with eight DAK-100 flasks, made of polytetrafluoroethylene (TFM).

The absorbance of the digestates was measured with a Cary 60 UV-Vis spectrophotometer from Agilent (Santa Clara, CA, USA), using a 10-mm quartz cuvette. As the obtained digestates presented a yellow and pale-yellow color, characteristic of the residual organic matter, we measured their absorbances at 415 nm, where the maximum absorbance of the yellow solutions occurred.

The sorption experiments were run in a batch mode using a laboratory roller mixer (model K45-8010) supplied by Kasvi (Curitiba, Brazil) and operated at 50 rpm. A Digimed (São Paulo, Brazil) pH meter, model DM-22, equipped with a combined glass electrode, was used to measure the pH of the solutions. Nitric acid was distilled in a sub-boiling distillation apparatus system (BSB-939-IR) from Berghof (Eningen, Germany).

### 2.2. Reagents and Solutions

All solutions used in this work were prepared with analytical grade reagents used without further purification. Ultrapure water with a resistivity of 18.2 MΩ·cm obtained in a Direct-Q 3 system from Millipore (Massachusetts, USA) was used in the preparation of all solutions.

The 1000 mg·L^−1^ Cd and Pb stock solutions were supplied by Tedia (Ohio, USA). Standard solutions with different concentrations were prepared through the adequate dilution of this stock solution just before use.

A 0.10 mol·L^−1^ Britton–Robinson buffer solution was prepared by dissolving 1.54 g of boric acid (Caledon, Ontário, Canadá), 1.40 mL of acetic acid (Vetec, Rio de Janeiro, Brazil), and 1.70 mL of phosphoric acid (Vetec, Rio de Janeiro, Brazil) in 100 mL of deionized water. Afterward, the pH of the solution was adjusted to 6.50 and 8.0 by adding NaOH (Vetec, Rio de Janeiro, Brazil) 6 mol·L^−1^, and the final volume was completed to 250 mL with deionized water in a volumetric flask. The same procedure was repeated to adjust the pH over a range from 2.0 to 9.0 during the study of the influence of pH.

The APDC solution was prepared by dissolving the solid reagent (Sigma-Aldrich, MO, USA) in ultrapure water just before use.

The nitric acid solutions used for the microwave digestion of PUF were prepared through the suitable dilution of the concentrated acid (Tedia, Ohio, USA) which had previously been distilled.

### 2.3. PUF Preparation

The commercial open-cell polyether-type polyurethane foam (22.5 mg·cm^−3^ density) was furnished by Guararapes Ltda., Brazil. It was cut into small pieces and shredded in a blender with deionized water and ethanol. Thereafter, the mixture was filtered and the obtained PUF was left to dry at ambient room temperature. The dry material was stored in a dark flask in order to protect it from light incidence.

### 2.4. General Extraction Procedure

The experiments were carried out by stirring 46.5 mL of sample, 1.0 mL of buffer solution (pH 7.0), 2.5 mL of 10% m/v APDC solution, and 400 mg of PUF for 30 min. Following its extraction, the PUF was separated by filtration and transferred to the microwave oven flask for the recovery of the analytes using 10 mL of 10.5 mol·L^−1^ HNO_3_ solution. The microwave digestion conditions were optimized and are presented in Table 1.

Following the microwave irradiation, the flasks were cooled to room temperature before opening, and then the obtained solution was diluted to 25 mL with ultrapure water for the determination of Cd and Pb by GF AAS.

### 2.5. Determination of the Metals in the PUF Digestates by GF AAS

The quantification of both Cd and Pb in the digestates or in the standard solutions was performed by injecting 20 *μ*L of the solutions into the graphite tube together with 10 *μ*L of a 500 mg·L^−1^ Pd solution as a chemical modifier. The measurements were always carried out in the integrated absorbance mode.  Table 2 presents the general temperature program that was applied.

## 3. Results and Discussion

### 3.1. Optimization of Microwave-Assisted PUF Digestion

The first step of this work involved setting up the best conditions for the microwave-assisted digestion of the polyurethane foam used for the sorption of the analytes since we had previously observed that this strategy was more efficient than common elution for the release of Cu(II) from a PUF solid phase [16]. For this purpose, the effects of both temperature and irradiation time were evaluated (see Table 1) for three concentrations of HNO_3_: 3.5, 7.0, and 10.5 mol·L^−1^. The digestion efficiency was monitored through the measurement of the absorbance of the solution at 415 nm, because the final digestates remained yellow (with maximum absorption at 415 nm) when no efficient digestion was achieved. All experiments were performed with 250 mg of PUF.

**Figure 1:**
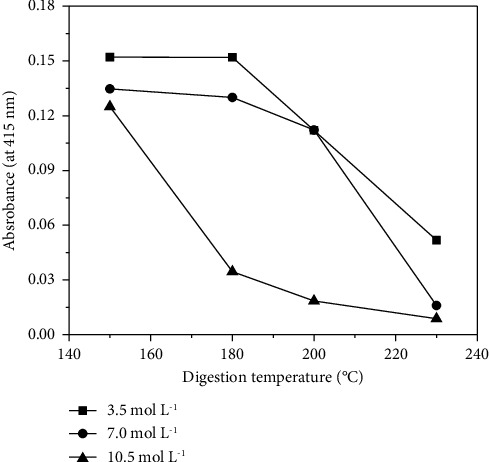
Influence of the digestion temperature on the absorbance of the digestates for the evaluation of digestion efficiency. Mass of PUF = 250 mg and irradiation time = 60 min.

The influence of the digestion temperature was evaluated in the range of 150–230°C. The results are shown in Figure 1. The profiles of the curves for the HNO_3_ concentrations of 3.5 and 7.0 mol·L^−1^ were very similar, with a significant decrease in the final absorbance of the solutions when temperatures of 200 or 230°C were used for the digestion. Certainly, this decrease indicated that more efficient mineralization of the organic PUF was observed at higher digestion temperatures. On the other hand, the profile of the curve for the 10.5 mol·L^−1^ HNO_3_ solution was different. In this case, the absorbance already decreased at 180°C, evidencing the additional effect of the acid concentration on the digestion process. Again, the lowest absorbance (maximum digestion efficiency) was verified at 230°C, and this temperature was selected for the heating program of the microwave oven.

**Figure 2:**
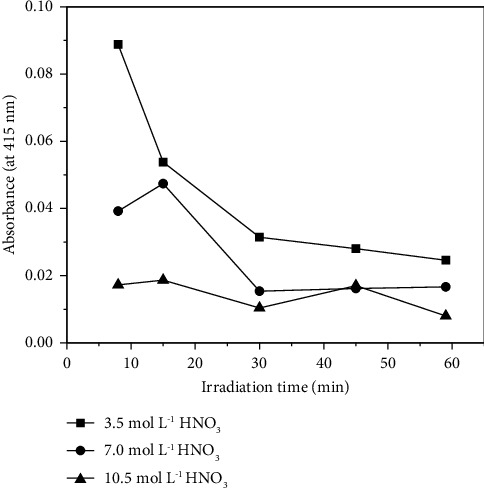
Influence of the irradiation time on the absorbance of the digestates for the evaluation of digestion efficiency. Mass of PUF = 250 mg and digestion temperature = 230°C.

Subsequently, the effect of the irradiation time on the absorbance of the digestates was investigated. This parameter was evaluated between 10 and 60 min, and the results are presented in Figure 2. As shown in Figure 2, the irradiation time had a more pronounced effect on the digestion with the lowest concentration (3.5 mol·L^−1^) of HNO_3_, whereas the absorbances were almost constant when a 10.5 mol·L^−1^ HNO_3_ solution was used for the digestion. Again, the final solutions with lower absorbances were verified when the HNO_3_ concentration was 10.5 mol·L^−1^, confirming that this solution was more efficient for PUF mineralization. For this reason, the nitric acid concentration of 10.5 mol·L^−1^ was chosen for the digestion method, combined with an irradiation time of 30 min, which was considered enough to promote a suitable degradation of PUF. It is important to reinforce that the digestion temperature was 230°C. These final conditions were used for the release of the analytes from PUF in all further experiments.

### 3.2. Construction of the Curves of Pyrolysis and Atomization

Before optimizing the extraction process, the program of temperature for the measurement of Cd and Pb in the digestates by GF AAS was evaluated. This study was carried out through the construction of the curves of pyrolysis and atomization and the evaluation of the temperature of the drying step for each metal using the solutions of Cd (1.0 *μ*g·L^−1^) and Pb (1.0 *μ*g·L^−1^) prepared through the spiking of digestates obtained from the digestion of 250 mg of PUF.

The temperature of the drying step was studied to avoid the rapid volatilization of the solvent, which could lead to the projection of the sample on the tube walls, resulting in low precision for the measurements [17]. To achieve this goal, the injected solution (sample or standard) was dried in two stages: (i) the first step was set to reach a temperature (95°C) just below the boiling point of the solvent (water, 100°C), and (ii) the second step was set to provide slower heating of the graphite tube from 95 to 140°C in 12 s (heating rate of 3.75°C s^−1^); once the latter temperature was achieved, the tube was maintained at this temperature for 20 s to ensure the elimination of any residual solvent present in the tube. The application of this heating pattern for the drying step was successful, and no splashing of the sample on the tube walls was observed.

**Figure 3:**
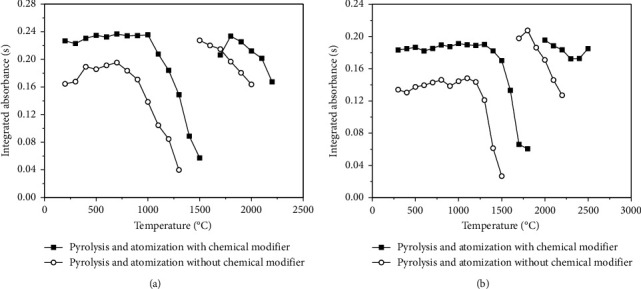
Curves of pyrolysis and atomization for (a) Cd(II) and (b) Pb(II) with and without the use of Pd as a chemical modifier. See details in the text.

Thereafter, two curves of pyrolysis and atomization were constructed for Cd and Pb, one using Pd as a chemical modifier and the other without Pd. The results are shown in Figure 3. Based on the temperature profiles obtained in this study, the temperatures of pyrolysis and atomization were set at 700 and 1800°C for Cd and 1000 and 2000°C for Pb, respectively. In addition, the use of a chemical modifier to reach a convenient thermal stabilization of the analytes is highly recommended for the determination of Cd and Pb by GF AAS [18]. As expected, the presence of Pd increased the analytical signals and allowed the use of higher pyrolysis temperatures, which caused a decrease in the background signal. Therefore, the use of Pd as a chemical modifier with a concentration of 500 mg·L^−1^ was mandatory for both analytes.

### 3.3. Study of the Extraction and Determination of Pb(II) and Cd(II)

#### 3.3.1. Effect of pH

Metallic cations are not naturally retained by polyurethane foam because of their high solubility in water and weak interaction with the foam structure. Therefore, it is important to change them into a form that can be sorbed by a solid phase. We have previously demonstrated that dithiocarbamate complexes of metallic cations have a great affinity with polyurethane foam [19, 20], being extracted through a mechanism similar to solvent extraction, in which PUF acts as a solid solvent [15]. In this work, we explored the formation of APDC complexes of Cd(II) and Pb(II) to create a “molecule” able to be retained by the foam. In this context, the sample pH is an important parameter to be controlled since APDC behaves as a weak acid in an aqueous medium and can be decomposed in an acidic one [21–23]. Additionally, the formation of metal hydroxyl complexes could influence the interaction of the analytes with APDC.

**Figure 4:**
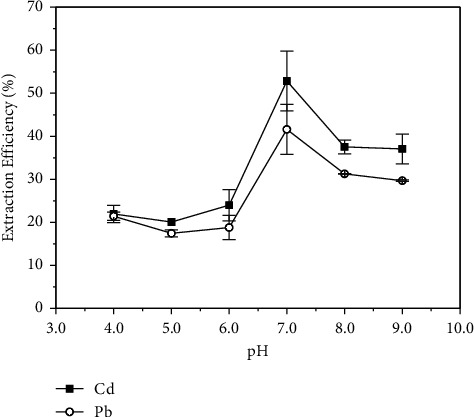
Influence of the pH on the extraction efficiency of (■) Cd and (○) Pb by polyurethane foam. Concentration of Cd = 0.5 mg·L^−1^, concentration of Pb = 2.0 mg·L^−1^, APDC concentration = 0.1% (m/v), shaking time = 30 min, and mass of PUF = 250 mg.

Cd(II) and Pb(II) can both bind with APDC, forming 1: 2 metal-ligand complexes through the electron donor sulfur atoms present in the molecule of the complexing agent [24, 25]. For this, it is more favorable to work with pH values greater than 4.0, since according to the APDC species distribution diagram, the unprotonated species will become predominant in the medium under these conditions. Therefore, the evaluation of the pH was carried out over a pH range of 4.0–9.0, using solutions containing 0.5 mg·L^−1^ and 2.0 mg·L^−1^ of Cd(II) and Pb(II), respectively, 250 mg of PUF, APDC concentrations of 0.1% (m/v), and 30 min of extraction time (the length of time that the system is maintained under shaking). The results are shown in Figure 4.

As expected, the results showed that the pH had a significant influence on the extraction of the analytes. Maximum extraction efficiency was observed at pH values of 7.0 for both Cd(II) and Pb(II). Therefore, this pH was chosen for the method. Below this value of pH, the concentration of unprotonated APDC was low, whereas at pH values exceeding 7.0, the occurrence of hydroxyl complexes of Cd(II) and Pb(II) probably interfered with the formation of metal-APDC complexes.

#### 3.3.2. Effect of the APDC Concentration

**Figure 5:**
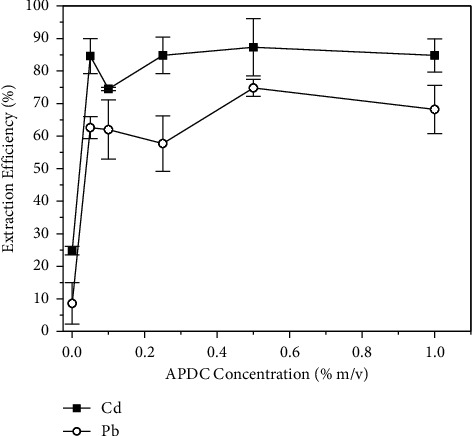
Influence of the APDC concentration on the extraction efficiency of (■) Cd and (○) Pb by polyurethane foam. Concentration of Cd = 0.1 mg·L^−1^, concentration of Pb = 0.5 mg·L^−1^, pH = 7.0, shaking time = 30 min, and mass of PUF = 250 mg.

Like the pH, the concentration of the complexing agent is responsible for controlling the availability of APDC in the medium to promote the formation of the metal-ligand complexes. Therefore, this parameter must also be optimized. To optimize the concentration of APDC in the solution, we used solutions containing 0.1 mg·L^−1^ and 0.5 mg·L^−1^ of Cd(II) and Pb(II), respectively, 250 mg of PUF, a pH of 7.0, and 30 min of extraction time. The results are shown in Figure 5.

As expected, the extraction efficiency of the analytes was very low without the addition of the complexing agent because Cd(II) and Pb(II) do not significantly interact with the polyurethane foam. A noticeable increase in the extraction efficiency was observed with the increase in the APDC concentration up to 0.5% (m/v) but no significant change was observed when the APDC concentration was increased to 1.0% (m/v). This indicates that the maximum complexation of the metallic cations had been reached, and the extraction would have been limited by other conditions such as the extraction time or the mass of the sorbent. To maximize the extraction of both analytes, we selected an APDC concentration of 0.5% (m/v) for the method.

#### 3.3.3. Effect of the Extraction Time (Shaking Time)

**Figure 6:**
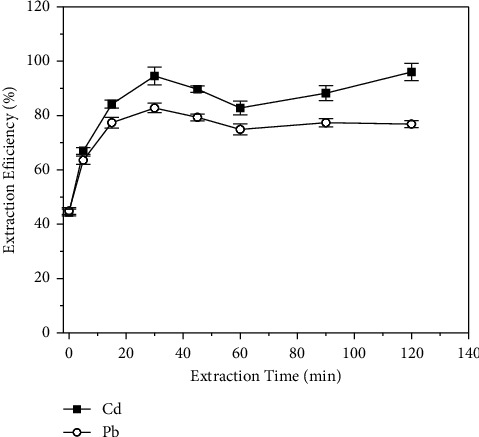
Influence of the extraction time on the extraction efficiency of (■) Cd and (○) Pb by polyurethane foam. Concentration of Cd = 0.1 mg·L^−1^, concentration of Pb = 0.5 mg·L^−1^, pH = 7.0, APDC concentration = 0.5% (m/v), and mass of PUF = 250 mg.

Another factor investigated in the present work was the extraction (shaking) time which controls the time of contact between the solid phase and the sample solution and can be very significant depending on the extraction kinetics. In this study, the two phases were maintained in contact by shaking using a horizontal roller mixer. The shaking time was varied from 5 to 120 min. For both analytes, the extraction efficiency increased with the increase in the extraction time up to 30 min, and beyond this value, this parameter no longer influenced the transference of the analytes from the liquid to the solid phase (Figure 6). Therefore, an extraction time of 30 min was set for the method. It is important to highlight that even for an extraction time of 30 min, quantitative extraction was not observed for Cd(II) or Pb(II), with extraction efficiencies never exceeding 90%. This result suggests that the process was probably limited by another factor such as the mass of the sorbent.

#### 3.3.4. Effect of Sorbent (PUF) Mass

**Figure 7:**
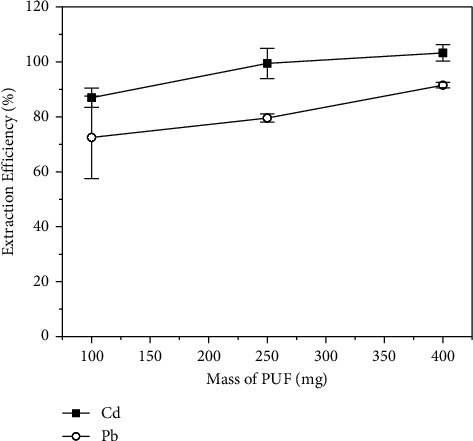
Influence of the sorbent mass on the extraction efficiency of (■) Cd and (○) Pb by polyurethane foam. Concentration of Cd = 0.1 mg·L^−1^, concentration of Pb = 0.5 mg·L^−1^, pH = 7.0, APDC concentration = 0.5% (m/v), and shaking time = 30 min.

The sorbent mass is an important parameter to be evaluated since it is directly related to the number of active sites available for the sorption of the analytes (as APDC complexes). Thus, this factor was evaluated with solutions containing 0.1 mg·L^−1^ of Cd(II) and 0.5 mg·L^−1^ of Pb(II), 0.5% (m/v) APDC solution, a pH of 7.0, and 30 min of shaking time. The results showed an improvement in the extraction efficiency of both analytes as the PUF mass was increased, reaching quantitative values (extraction efficiency greater than 90%) for both analytes when 400 mg of PUF were used (Figure 7). Greater amounts of adsorbent were not tested because of the capacity of the microwave oven to digest the foam. Therefore, a mass of 400 mg was selected for further studies considering a better extraction efficiency of the analytes as well as the total digestion of the material using the microwave-assisted method.

### 3.4. Selectivity of the Proposed Method

The sorption of the analytes by PUF in the form of metal-APDC complexes was evaluated in the presence of the Zn(II), Fe(III), Ca(II), Mn(II), and Cu(II). We selected cations that may be complexed with APDC as potential interferents. Interference was considered to have occurred when a signal difference greater than 10% was observed.

The interfering ions were added individually at concentrations ranging from 0.5 to 5 mg·L^−1^ to a solution containing 0.1 mg·L^−1^ and 0.5 mg·L^−1^ of Cd(II) and Pb(II), respectively. The results obtained in this study showed that the presence of Cu(II), Mn(II), Ca(II), and Fe(III) caused a variation lower than 10% in the signal of Cd(II), which was considered relatively insignificant. On the other hand, the presence of Zn(II) resulted in a decrease of the Cd(II) signal between 23% and 31% as the concentration of the interfering cation was increased. Mn(II) did not interfere with the Pb(II) sorption within the studied range, whereas 1 mg·L^−1^ of Zn(II) and Fe(III) caused a decrease in its extraction efficiency by 35% and 17%, respectively. The presence of Ca(II) and Cu(II) at 1 mg·L^−1^ reduced Pb(II) extraction by 21% and 15%, respectively. However, it is important to note that the concentrations of possible interferents in the samples should not reach the mg·L^−1^ level.

### 3.5. Figures of Merit of the Method

The figures of merit were obtained and calculated from the analytical curves constructed over concentration ranges of 0.06-3 *μ*g·L^−1^ and 1.8–40 *μ*g·L^−1^ for Cd and Pb, respectively (Table 3).

Good sensitivity was obtained for the determination of metals using the proposed method. The analytical curves presented a satisfactory linearity with the coefficients of determination (*r*^2^) always exceeding 0.99. The estimated limits of detection and quantification were compared with the maximum values allowed by CONAMA, showing that the analytes could be quantified with the developed method when present in concentrations below the permitted limits of 10 *μ*g·L^−1^ for Pb(II) and 1 *μ*g·L^−1^ for Cd(II). The intermediary precision was estimated through the analysis of the SPW_4_ sample (the only sample that contained both Cd and Pb) on three different days which yielded values of 6.1 and 7.3% for Cd and Pb, respectively.

### 3.6. Determination of Cd and Pb in Swimming Pool Waters

The developed method was used for the preconcentration and determination of Pb(II) and Cd(II) concentrations in swimming pool water samples using GF AAS. The results are listed in Table 4. Samples SPW_1_, SPW_2_, and SPW_4_ presented the concentrations of Cd(II) below the maximum permitted in the Brazilian legislation (1.0 *μ*g·L^−1^ for Cd(II)). On the other hand, sample SPW_3_ presented a mean concentration of Cd(II) numerically above the limit, but not statistically different from it. The concentrations of Pb(II) in samples SPW_1_, SPW_2_, and SPW_3_ were between the limits of detection and quantification estimated for the method, while the sample SPW_4_ yielded a Pb(II) concentration above the legal limit (10.0 *μ*g·L^−1^).

The accuracy of the method was tested through the application of a recovery test. As shown in Table 4, satisfactory recovery rates (82 to 105%) were obtained in the analysis of the spiked samples. These results indicate that the proposed method can be reliably used for the determination of these metal ions in the real matrices of swimming pool waters.

## 4. Conclusion

The developed method presented a good analytical performance and can be considered as an option for the determination of Cd(II) and Pb(II) in swimming pool waters using GF AAS. Moreover, we suggest that this method could be used in the analysis of other types of water. The use of APDC as a complexing agent for the sorption of metals onto PUF was shown to be efficient, and the procedure was simple and fast and used a low-cost material such as PUF.

The total digestion of the loaded sorbent (500 mg) was achieved by using diluted solutions of HNO_3_ (10.5 mol·L^−1^) and 30 min of microwave irradiation at 230°C. In these conditions, the release of Cd(II) and Pb(II) from the PUF was successfully achieved, and the digestates could be employed for their determination by atomic absorption spectrometry. We believe that the developed method can be used in other procedures as an alternative to conventional elution processes.

The concentration of both Cd(II) and Pb(II) in the analyzed samples was always below the limits imposed by the Brazilian law for both Cd and Pb, except for one sample that presented Pb(II) concentration higher than that indicated in the legislation.

## Figures and Tables

**Table 1:** Microwave oven program used in the wet acid digest of polyurethane foam with a 10.5 mol·L^−1^ HNO_3_ solution.

Step	T (°C)	Ramp (°C·s^−1^)	Irradiation time (min)
1	100	1	2
2	230^a^	2	30^a^
3	50	1	1
4	50	1	1
5	50	1	1

^a^Values set after the study of the microwave-assisted digestion conditions (see text for details).

**Table 2:** Temperature program employed in the determination of Cd and Pb in the digestates of polyurethane foam by GF AAS.

Step	T (°C)	Ramp (s)	Hold (s)	Ar flow rate (L·min^−1^)
Drying I	95	10.0	—	0.3

Drying II	140	12.0	20.0	0.3

Pyrolysis^a^	700 (Cd)	5.0	5.0	0.3
	1000 (Pb)			

Atomization^a^	1800 (Cd)	2.0	1.0 (Cd)	0.0
	2000 (Pb)		2.0 (Pb)	

Cleaning	2000 (Cd)	5.0	—	0.3
	2100 (Pb)			

^a^Optimum temperatures of pyrolysis and atomization were set by analyzing the profile of the respective curves (see text for details).

**Table 3:** Analytical figures of merit for the determination of Cd and Pb in swimming pool waters by GF AAS after preconcentration on polyurethane foam.

Parameter	Pb(II)	Cd(II)
Concentration range	1.8–40 *μ*g·L^−1^	0.06–3 *μ*g·L^−1^
LOD	0.5 *μ*g·L^−1^	0.02 *μ*g·L^−1^
LOQ	1.8 *μ*g·L^−1^	0.06 *μ*g·L^−1^
Calibration equation	*A* = 0.0069 (Pb (*μ*g L^−1^)) + 0.0047	*A* = 0.16 (Cd (*μ*g L^−1^)) + 0.0018
*r*^2^	0.9976	0.9996
Intermediary precision	7.3%	6.1%

**Table 4:** Results obtained for recoveries of spike additions of lead and cadmium in water samples.

Sample	Pb concentration (*μ*g L^−1^)		Recovery (%)	Cd concentration (*μ*g L^−1^)		Recovery (%)
SPW_1_	0	<LOQ	—	0	0.22 ± 0.04	—
	10.0	8.3 ± 0.2	83 ± 2	1.0	1.14 ± 0.16	92 ± 16

SPW_2_	0	<LOQ	—	0	0.44 ± 0.09	—
	10.0	9.2 ± 0.4	92 ± 4	1.0	1.37 ± 0.04	93 ± 3

SPW_3_	0	<LOQ	—	0	1.02 ± 0.04	—
	10.0	8.8 ± 0.3	88 ± 3	1.0	2.05 ± 0.05	103 ± 3

SPW_4_	0	11.4 ± 2	—	0	0.86 ± 0.03	—
	10.0	21.9 ± 0.8	105 ± 4	1.0	1.68 ± 0.04	82 ± 2

## Data Availability

The data that support the findings of this study are available from the corresponding author upon reasonable request.
